# Hyperglycemia, inflammatory response and infarct size in obstructive acute myocardial infarction and MINOCA

**DOI:** 10.1186/s12933-021-01222-9

**Published:** 2021-02-02

**Authors:** Pasquale Paolisso, Alberto Foà, Luca Bergamaschi, Francesco Donati, Michele Fabrizio, Chiara Chiti, Francesco Angeli, Sebastiano Toniolo, Andrea Stefanizzi, Matteo Armillotta, Paola Rucci, Gianmarco Iannopollo, Gianni Casella, Cinzia Marrozzini, Nazzareno Galiè, Carmine Pizzi

**Affiliations:** 1 Unit of Cardiology, Department of Experimental, Diagnostic and Specialty Medicine-DIMES, University of Bologna, Via Giuseppe Massarenti 9, Bologna, 40138 Italy; 2 Division of Hygiene and Biostatistics, Department of Biomedical and Neuromotor Sciences, Alma Mater Studiorum, University of Bologna, Bologna, Italy; 3 Unit of Cardiology, Maggiore Hospital, Bologna, Italy

**Keywords:** Hyperglycemia, Inflammation, Infarct size, MINOCA, Obstructive acute myocardial infarction

## Abstract

**Background:**

Hyperglycemia has been associated with increased inflammatory indexes and larger infarct sizes in patients with obstructive acute myocardial infarction (obs-AMI). In contrast, no studies have explored these correlations in non-obstructive acute myocardial infarction (MINOCA). We investigated the relationship between hyperglycemia, inflammation and infarct size in a cohort of AMI patients that included MINOCA.

**Methods:**

Patients with AMI undergoing coronary angiography between 2016 and 2020 were enrolled. The following inflammatory markers were evaluated: C-reactive protein, neutrophil-to-lymphocyte ratio (NLR), platelet-to-lymphocyte ratio (PLR) and neutrophil-to-platelet ratio (NPR). Myocardial infarct size was measured by peak high sensitivity troponin I (Hs-TnI) levels, left-ventricular-end-diastolic-volume (LVEDV) and left ventricular ejection fraction (LVEF).

**Results:**

The final study population consisted of 2450 patients with obs-AMI and 239 with MINOCA. Hyperglycemia was more prevalent among obs-AMI cases. In all hyperglycemic patients—obs-AMI and MINOCA—NLR, NPR, and LPR were markedly altered. Hyperglycemic obs-AMI subjects exhibited a higher Hs-TnI (p < 0.001), a larger LVEDV (p = 0.003) and a lower LVEF (p < 0.001) compared to normoglycemic ones. Conversely, MINOCA patients showed a trivial myocardial damage, irrespective of admission glucose levels.

**Conclusions:**

Our data confirm the association of hyperglycemic obs-AMI with elevated inflammatory markers and larger infarct sizes. MINOCA patients exhibited modest myocardial damage, regardless of admission glucose levels.

## Background

Hyperglycemia frequently occurs in patients admitted for acute myocardial infarction (AMI), irrespective of a previously documented diabetes mellitus (DM) [1]. In particular, approximately 10% to 20% of non-diabetic AMI patients have significant hyperglycemia [2]. Recent data demonstrated that hyperglycemia is associated with an increased risk of major adverse cardiovascular events (MACE) [2, 3]. Additionally, amongst patients with large infarct sizes, hyperglycemia has been identified as a prognostic marker both in patients with and without diabetes [4–6].

So far, it is unexplained whether elevated admission high glucose levels (aHGL) are a marker of more extensive myocardial damage or a prognostic risk factor in patients with AMI [7].

In order to unravel the association between aHGL and the increased risk of adverse cardiovascular events, several potential explanations have been suggested. Systemic immune activation, modification of platelet function and thrombotic-fibrinolysis system, abnormal autonomic tone, increased oxidative stress, endothelial dysfunction and impaired myocardial contractility seem to play a role in myocardial damage [8–10].

Etiopathogenetic mechanisms underlying hyperglycemia in the acute phase of myocardial infarction have not been fully elucidated. Blood glucose levels can be transiently elevated either as a stress response to acute illness (stress hyperglycemia), resulting from an inflammatory and adrenergic adaptation to ischemic injury (release of catecholamines and steroids and glycogenolysis induction), or as a reflection of an underlying abnormal glucometabolic state.

In the context of AMI, a series of ischemia-mediated pathophysiological events occur, generating an intense inflammatory response. Neutrophils are the first leukocytes detected in infarcted areas, followed by monocytes and lymphocytes, which, releasing proteo-enzymes and cytokines, phagocytize necrotic debris and promote the subsequent proliferative process [11]. Additionally, activated platelets, besides acutely precipitating vascular obstruction, further amplify the inflammatory response interacting with neutrophils, monocytes and lymphocytes. Therefore, the role of inflammatory cells is not limited to the acute ischemic event but drives the chronic atherosclerotic process as well.

Recent accumulating evidence suggests that neutrophil-to-lymphocyte ratio (NLR), platelet-to-lymphocyte ratio (PLR) and neutrophil-to-platelet ratio (NPR) might be considered as biomarkers of systemic inflammation and have been associated with poor clinical outcomes in various cardiovascular diseases, including acute coronary syndromes [12–15]*.*

The link between aHGL and inflammation is nowadays well established as it is the prognostic role of hyperglycemia in the context of AMI with obstructive coronary artery disease (obs-AMI). On the other hand, the relationship between hyperglycemia and inflammatory response in myocardial infarction with non-obstructive coronary arteries (MINOCA) is still poorly explored.

Our study sought to investigate the association between hyperglycemia and inflammatory status as well as myocardial damage/severity in patients with obs-AMI versus MINOCA.

## Materials and methods

### Patients

All consecutive patients hospitalized for AMI (Policlinico Sant’Orsola-Malpighi Hospital and Maggiore Hospital, Bologna—Italy) who underwent coronary angiography (CAG) within the first 72 h from admission between January 2016 and March 2020 were included in the study. AMI was diagnosed in the presence of an increase and/or decrease of cardiac biomarker (troponin I high sensitivity—Tn I Hs) with at least one value above the 99th percentile upper reference limit associated with one of the following: symptoms of ischemia, new or presumed new significant ST-segment–T wave changes or new left bundle branch block, development of pathological Q waves in the EKG, and imaging evidence of new loss of viable myocardium or new regional wall motion abnormality [16, 17]. MINOCA was diagnosed according to the 2016 ESC MINOCA Position Paper criteria [18, 19]. Patients whose admission glycemia was not available were excluded from the study. Other exclusion criteria were severe valvular heart disease, prosthetic heart valves, severe anaemia, major acute bleeding, pulmonary embolism, fever (38 °C), hypertensive crisis, chronic renal failure (glomerular filtration rate < 30 mL/min/1.73 m2), autoimmune diseases, malignancies or ongoing cardiotoxic medications, and congenital heart disease.

Data were collected as part of an approved multicenter observational study called “AMIPE: Acute Myocardial Infarction, Prognostic and Therapeutic Evaluation” (ClinicalTrials.gov Identifier: NCT03883711). The present study was conducted according to the principles of the Declaration of Helsinki; all patients were informed about their participation in the registry and provided informed consent for the anonymous publication of scientific data.

### Inflammatory biomarkers and infarct size detection

The inflammatory response was evaluated using the following parameters: NLR, NPR, PLR, C-Reactive Proteine. In particular, NLR is the ratio of neutrophil and lymphocyte counts, NPR is the ratio of neutrophil and platelet counts, and PLR is obtained by dividing the platelet count by the lymphocytes. The other laboratory parameters were determined according to standard protocols.

For all patients, blood for hs-TnI evaluation was drawn at the moment of hospital admission and every 3–6 h thereafter for the following 24 h. The hs-TnI peak was considered the highest value before its fall. Comprehensive echocardiographic studies, including Doppler studies, were performed according to the current European recommendations [20].

All patients underwent 2D-echocardiogram before discharge, approximately 3 days after hospitalization. All studies were performed by experienced operators using Philips EPIQ and Affiniti ultrasonography machines. At least 3 consecutive beats were recorded for each view and all images were stored for offline analysis. Left ventricular ejection fraction (LVEF) was calculated with the biplane Simpson’s method acquiring volumes in both 4- and 2-chamber views, according to the European Association of Cardiovascular Imaging Guidelines [20]. Myocardial infarct size was also estimated using the left ventricular end-diastolic volume (LVEDV) and the left ventricular ejection fraction (LVEF).

### Blood glucose and definition of hyperglycemia

Blood glucose levels were assessed at admission as part of the standard evaluation. Pre-existing DM was defined as known DM at the time of hospitalization irrespective of the therapeutic management (either diet and lifestyle measures alone or additional administration of oral glucose-lowering medication and insulin) [21]. According to the American Heart Association Scientific Statement, patients were categorised based on admission glucose levels as follows: normoglycemia < 140 mg/dl and hyperglycemia ≥ 140 mg/dl [2].

### Statistical analysis

**Fig. 1:**
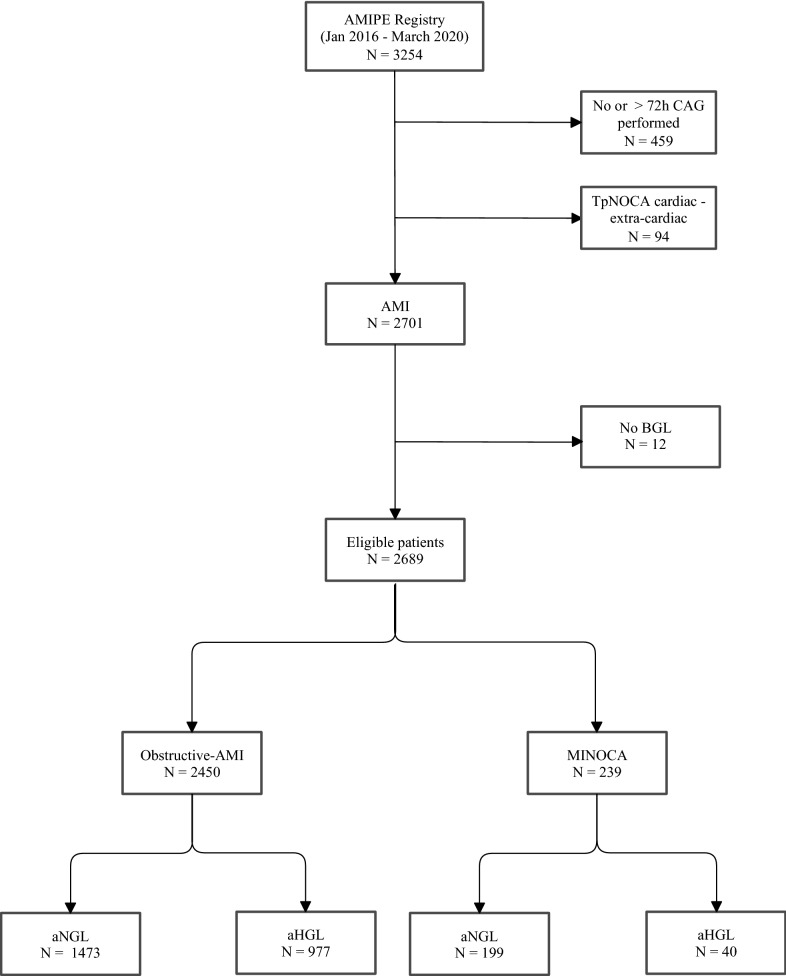
Flow chart Study. *CAG* coronary angiography, *AMI* acute myocardial infarction, *TpNOCA* troponin-positive non-obstructive coronary arteries, *Obs-AMI* obstructive myocardial infarction, *MINOCA* myocardial infarction with non-obstructive coronary arteries, aBGL admission blood glucose level, *aHGL* admission high glucose level, *aNGL* admission normal glucose level

We analyzed the correlation of inflammatory and infarct size markers with hyperglycemia at hospital admission in patients with obs-AMI and in those with MINOCA. To this purpose, we first assessed the distribution of laboratory parameters using Shapiro-Wilks test and the homogeneity of variance using Levene’s test. We then compared laboratory parameters and infarct sizes between patients with or without hyperglycemia using Mann–Whitney U test or Student's t-test as appropriate. Categorical variables were compared between groups using χ [2] test. Lastly, we investigated differences in inflammatory biomarkers and infarct size of hyperglycemia in obstructive-AMI (obs-AMI) and MINOCA patients using univariate logistic regression models. The significance level was set to p < 0.05, and all analyses were performed using Stata 13.1 (Stata Corp., College Station, Texas, 2013) and IBM SPSS, version 25.0 (Fig. 1).

## Results

**Fig. 2:**
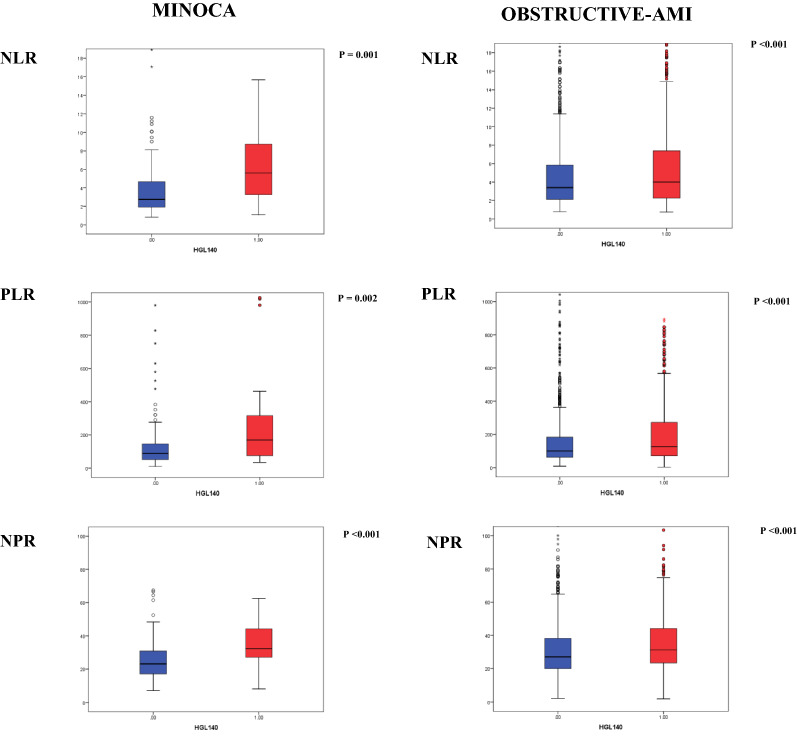
Inflammatory markers in obstructive Acute Myocardial Infarction and Non-obstructive acute myocardial infarction. The blue colour denotes normoglycemic patients; the red colour represents hyperglycemic patients. *AMI* acute myocardial infarction, *MINOCA* non-obstructive acute myocardial infarction, *NLR* Neutrophil-to-lymphocyte ratio, *NPR* Neutrophil-to-platelet ratio, *PLR* Platelet-to-lymphocyte ratio

A total of 2795 patients with suspected AMI who underwent coronary angiography within 72 h of symptom onset were evaluated. Following diagnostic workup, 94 patients were excluded from the study due to a non-ischemic troponin elevation, including 16 myocarditis and 52 Tako-tsubo syndromes. Among cases diagnosed with AMI, 12 patients (10 obs-AMI and 2 MINOCA) were excluded because blood glucose level at hospital admission was not available. The final study population consisted of 2450 patients with obs-AMI and 239 with MINOCA. Clinical and angiographic characteristics of obs-AMI subjects as well as etiopathological causes of MINOCA are shown in Table 1. Both groups were divided according to the presence of aHGL. Demographic and clinical characteristics are shown in Table 2. Overall, admission aHGL was noticed in 1017 patients (37.8%), more frequently in patients with obs-AMI compared to MINOCA (40% versus 16.7%; p < 0.001). The parameters of infarct size and myocardial damage/inflammation of each group are presented in Table 3 and Fig. 2.

**Table 1:** Clinical and angiographic characteristics of obstructive-AMI and MINOCA patients

	Obstructive AMI N = 2450		MINOCA N = 239		
	STEMI N = 1116	NSTEMI N = 1334		STEMI N = 28	NSTEMI N = 211
Lesion Location			Causes		
LM lesion, n (%)	47 (4.2)	195 (14.6)	Epicardial coronary spasm	1 (3.6)	13 (6.2)
LAD lesion, n (%)	954 (85.4)	988 (74)	SCAD	9 (32.1)	26 (12.3)
LCx lesion, n (%)	412 (36.9)	520 (38.9)	Coronary embolism	4 (14.3)	0 (0)
RC lesion, n (%)	744 (66.7)	759 (56.9)	Atherosclerotic plaque disruption (type I)	5 (17.9)	31 (14.7)
			Supply–demand mismatch (type II)	9 (32.1)	141 (66.8)
Number of Vessels			Number of vessels with stenosis		
LM, n (%)	37 (3.3)	148 (11.1)	LM (1–20%), n (%)	4 (14.3)	1 (0.5)
1 Vessel, n (%)	667 (59.8)	459 (34.4)	1 Vessel (20–49%), n (%)	3 (10.7)	31 (14.6)
2 Vessels, n (%)	303 (27.2)	383 (28.7)	2 Vessels (20–49%), n (%)	3 (10.7)	13 (6.2)
3 Vessels, n (%)	109 (9.7)	344 (25.8)	3 Vessels (20–49%), n (%)	0 (0)	3 (1.4)

*AMI* Acute Myocardial Infarction, *LAD* Left Anterior Descending artery, *LCx * Left Circumflex, *LM* Left Main, *MINOCA* myocardial Infarction with Non-Obstructive Coronary Arteries,* NSTEMI * Non-ST-segment elevation myocardial infarction, *RC* Right Coronary artery, *STEMI* ST-segment elevation myocardial infarction, *SCAD* Spontaneous coronary artery dissection

**Table 2:** Demographic, clinical, laboratory findings and treatment of obstructive-AMI and MINOCA patients, according to admission to hyperglycemia

	Obstructive-AMI N = 2450			MINOCA N = 239		
	aHGL N = 977	aNGL N = 1473	*p-value*	aHGL N = 40	aNGL N = 199	*p-value*
Age, years, median (IQR)	72.0 (62.0–80.0)	68.0 (58.0–78.0)	< 0.001	74 (67–81)	68 (53 – 77)	0.001
Gender Female, n (%)	280 (28.7)	383 (26)	0.1	28 (70)	129 (64.8)	0.5
BMI Kg/m^2^, median (IQR)	26.8 (24.2–30.3)	26.2 (23.9–29.0)	0.001	25.9 (22.8–29.2)	25.6 (22.4 – 28.2)	0.6
Cardiovascular risk factors						
Current/past smoking, n (%)	547 (56.3)	908 (62.4)	0.007	13 (32.5)	88 (44.7)	0.1
Hypertension, n (%)	720 (74.2)	967 (65.9)	< 0.001	30 (75)	129 (65.2)	0.2
Dyslipidemia, n (%)	595 (61.3)	898 (61.2)	0.9	21 (52.5)	123 (61.8)	0.3
Type-2 diabetes, n (%)	477 (48.8)	113 (7.7)	< 0.001	12 (30.0)	11 (5.5)	< 0.001
Medical history						
Previous AMI, n (%)	238 (24.5)	290 (19.8)	0.006	2 (5.4)	18 (9.8)	0.4
Previous stroke, n (%)	80 (8.2)	79 (5.4)	0.005	2 (5.0)	11 (5.5)	0.8
COPD, n (%)	122 (12.5)	152 (10.3)	0.09	5 (12.5)	21 (10.6)	0.7
PAD, n (%)	103 (10.6)	85 (5.8)	< 0.001	2 (5)	5 (2.5)	0.4
Clinical presentations						
Angina, n (%)	813 (83.7)	1337 (91)	< 0.001	28 (70)	170 (85.4)	0.02
HR, median (IQR)	81 (70–97)	75 (65–88)	< 0.001	95 (76–134)	80 (66 – 93)	< 0.001
SBP, median (IQR)	140 (120–160)	140 (120–160)	0.5	140 (118–160)	140 (120 – 155)	0.7
DBP, median (IQR)	80 (70–90)	80 (70–90)	0.3	80 (70–85)	80 (70 – 90)	0.4
Atrial fibrillation, n (%)	103 (10.6)	93 (6.4)	< 0.001	13 (32.5)	14 (7.1)	< 0.001
STEMI, n (%)	468 (47.9)	648 (43.9)	0.057	5 (12.5)	23 (11.6)	0.8
Laboratory parameters						
Hemoglobin g/dL, median (IQR)	13.6 (12.1–15.0)	14.0 (12.7–15.1)	0.001	13.2 (12.1–14.8)	13.4 (12.1 – 14.5)	0.9
Admission BGL level mg/dL, median (IQR)	183 (157–238)	111 (99–122)	< 0.001	183 (154–227)	104 (93 – 117)	< 0.001
Discharge BGL level, mg/dl, median (IQR)	114 (97–145)	98 (85–112)	< 0.001	105 (92–127)	97 (85.0 – 111)	0.02
HbA1c, mmol/mol, median (IQR)	47 (40–60)	37 (34–40)	< 0.001	40 (37–50)	36 (32 – 40)	0.003
Creatinine mg/dl, median (IQR)	1.0 (0.9–1.3)	0.9 (0.8–1.1)	< 0.001	1.0 (0.7–1.2)	0.8 (0.7 – 1.0)	0.04
C-TOT, mg/dL median (IQR)	181 (149–216)	192 (161–222)	< 0.001	169 (151–205)	197 (167 – 224)	0.03
C-LDL, mg/dL median (IQR)	111 (85–139)	121 (93–149)	< 0.001	97 (84–127)	118 (97 – 144)	0.04
Tryglicerides, median (IQR)	116 (84–165)	112 (83–153)	0.02	116 (89–143)	111 (80 – 153)	0.8
Admission medical therapy						
Aspirin, n (%)	374 (38.6)	501 (34.2)	0.03	8 (20)	50 (25.1)	0.5
P2Y12 Inhibitor,s n (%)	99 (10.2)	110 (7.5)	0.02	2 (5)	9 (4.5)	0.9
Beta-blockers, n (%)	401 (41.4)	520 (35.6)	0.004	17 (42.5)	60 (30.2)	0.1
RAAS inhibitors, n (%)	504 (52)	659 (45.1)	0.002	23 (57.5)	64 (32.2)	0.002
Statins, n (%)	297 (30.7)	395 (27)	0.048	15 (37.5)	51 (25.6)	0.1
Admission glucose-lowering agents						
Insulin sensitizers (metformin), n (%)	259 (31.4)	69 (4.3)	< 0.001	5 (14.7)	8 (4)	0.01
Insulin providers (sulfonylureas), n (%)	160 (19.4)	37 (2.3)	< 0.001	3 (8.8)	2 (1.0)	0.004
DPP-4 Inhibitors, n (%)	29 (3.5)	6 (0.4)	< 0.001	1 (2.9)	1 (0.5)	0.1
GLP-1 Agonist, n (%)	7 (0.8)	2 (0.1)	0.02	0	0	0.99
SGLT-2 Inhibitors, n (%)	3 (0.4)	2 (0.1)	0.06	0	0	0.99
Insulin, n (%)	120 (14.6)	27 (1.7)	< 0.001	2 (5.9)	0	0.001
PCI total, n (%)	809 (83)	1213 (82.8)	0.9	0	0	0.99
PCI NSTEMI, n (%)	325 (75.9)	586 (76.4)	0.85	0	0	0.99

Continuous variables are presented as median (IQR) while categorical ones as n (%). *AMI*  acute myocardial infarction, *MINOCA*  myocardial infarction with non-obstructive coronary arteries, *Obs-AMI*   obstructive acute myocardial infarction, *aHGL*  admission High Glucose Level, *aNGL*   admission normal glucose level, *BMI  *body max index, *COPD  *chronic obstructive pulmonary disease, *HR*  heart rate, *SBP*  systolic blood pressure, *DBP*  diastolic blood pressure, *STEMI  *ST-segment Elevation Myocardial Infarction, *BGL  *blood glucose level, *HbA1c*  glycated hemoglobin, *C-TOT* = total cholesterol, *LDL-c*   LDL cholesterol, *RAAS  *Renin–angiotensin–aldosterone system, *DPP-4*  dipeptidyl peptidase 4, *GLP-1*  glucagon-like peptide 1, *SGLT-2*  Sodium glucose co-transporter 2, *PCI*  percutaneous coronary intervention

The last column shows the comparison between hyperglycemic obstructive-AMI and MINOCA patients

**Table 3:** Inflammation markers and infarct size in Obstructive-AMI and MINOCA patients, according to admission hyperglycemia

	Obstructive-AMI N = 2450			MINOCA N = 239			aHGL Obs-AMI vs MINOCA
	aHGL N = 977	aNGL N = 1473	*p-value*	aHGL N = 40	aNGL N = 199	*p-value*	*p-value*
Inflammation markers (admission—T0)							
WBC N/µl, median (IQR)	10.5 (8.1–13.3)	9.2 (7.4–11.6)	< 0.001	10.4 (8.1–14.9)	8.1 (6.6–10.1)	< 0.001	ns
Neutrophil N/µl, median (IQR)	7197 (5385–10,249)	6228 (4678–8623)	< 0.001	7933 (5637–11,443)	5305 (4053–7361)	< 0.001	ns
Lymphocyte N/µl, median (IQR)	1710 (1187–2530)	1787 (1312–2496)	0.2	1736 (1165–2164)	1840 (1385–2310)	0.3	ns
PLTs count × 10^9^ per L, median (IQR)	233 (193–282)	228 (189–275)	0.1	234 (195–287)	239 (200–289)	0.8	ns
CRP mg/dL, median (IQR)	0.5 (0.2–1.4)	0.4 (0.2–0.8)	< 0.001	0.5 (0.2–1.6)	0.3 (0.1–0.7)	0.04	ns
Inflammation markers (24 h—T1)							
WBC N/µl, median (IQR)	9.7 (7.9–12.3)	8.7 (7.0–10.9)	< 0.001	7.9 (7.0–10.9)	7.4 (6.2–8.8)	0.04	0.008
Neutrophil N/µl, median (IQR)	6935 (5344–9392)	5881 (4425–7730)	< 0.001	5505 (3851–9122)	4590 (3519–6527)	0.08	0.026
Lymphocyte N/µl, median (IQR)	1688 (1187 – 2219)	1839 (1376 –2407)	< 0.001	2012 (1042 – 2419)	1817 (1367 – 2304)	0.9	ns
CRP mg/dL, median (IQR)	1.1 (0.4 – 4.3)	0.7 (0.3 – 1.8)	< 0.001	1.0 (0.4 – 2.3)	0.5 (0.2 – 1.0)	0.06	ns
Infarct size							
LVEDV ml, median (IQR)	108 (84 – 135)	100 (83 – 121)	0.003	80 (70 – 121)	89 (74 – 107)	0.7	0.016
LV EF %, median (IQR)	47 (40 – 56)	55 (45 – 60)	< 0.001	59 (50 – 61)	60 (53 – 62)	0.8	< 0.001
Peak hs Troponin ng/L, median (IQR)	6556 (959 – 35,531)	2936 (576 – 18,164)	< 0.001	369 (133 – 901)	461 (113 – 1661)	0.5	< 0.001

Continuous variables are presented as median (IQR) while categorical ones as n (%).*AMI*  Acute myocardial infarction, *MINOCA*  Myocardial infarction with non-obstructive coronary arteries, *Obs-AMI*   Obstructive acute myocardial infarction, *aHGL*  Admission high glucose level, *aNGL*   Admission normal glucose level, *WBC*  White blood cell,* PLTs*  Platelets, *NLR*  Neutrophil-to-lymphocyte ratio, *PLR*  Platelet-to-lymphocyte ratio, *NPR*  Neutrophil-to-platelet ratio*, CRP*  C-reactive protein, *LVEDV*  Left ventricular end diastolic diameter; *LVEDV * Left ventricular end diastolic volume, *LVEF *Left ventricular ejection fraction, *Hs*  High sensitivity

### Obstructive-AMI: hyperglycemic vs normoglycemic patients

Over the 2450 patients with obs-AMI, hyperglycemia at admission was detected in 977 (40%) while no cases of hypoglycemia were observed. Notably, among hyperglycemic patients, a known T2DM was recorded in approximately half cases while less than 10% of normoglycemic subjects were diabetic. Hyperglycemic patients exhibited a worse cardiovascular risk profile and more comorbidities compared to normoglycemic ones. In fact, they were older, generally overweight, with a higher prevalence of hypertension and a history of cardiovascular events. As expected, a hyperglycemic status reflected an underlying altered glycol-lipid profile and was associated with a greater comorbidity burden, such as atrial fibrillation and chronic lung disease. Over 90% of normoglycemic obs-AMI patients presented with typical angina, while the percentage dropped to 83% among hyperglycemic patients (p < 0.001). Lastly, STEMI diagnosis at admission was similar between subgroups.

### MINOCA: hyperglycemic vs normoglycemic patients

Among the 239 patients diagnosed with MINOCA, only 16.7% exhibited a hyperglycemic state at admission, and no cases of hypoglycemia were observed. Hyperglycemic patients were significantly older, with a higher prevalence of hypertension. Similarly to the obstructive cohort, hyperglycemic cases showed a worse metabolic profile, with higher cholesterol levels and a greater prevalence of T2DM. Interestingly, the glycemic status did not affect the history of cardiovascular events or the prevalence of comorbidities, except for atrial fibrillation which was more frequent among hyperglycemic patients. Again, typical angina was frequently observed among normoglycemic patients, while 35% of hyperglycemic subjects had a different clinical presentation (p = 0.004). STEMI was equally diagnosed among cohorts.

### Impact of admission hyperglycemia on inflammatory markers and infarct size: obstructive-AMI vs MINOCA patients

In obs-AMI patients, total white blood cell count, neutrophils, platelets, CRP and peak troponin I levels were significantly higher in aHGL group compared to normoglycemic cases (Table 3). Moreover, all inflammatory parameters (NLR, NPR and LPR) were markedly altered in hyperglycemic subjects, both at admission and after 24 h (Fig. 2 and Table 3). Additionally, these patients exhibited a greater LVEDV and a lower LVEF compared to normoglycemic ones. In the MINOCA cohort, inflammatory markers at admission were significantly higher in aHGL group compared to normoglycemic patients while no differences were observed after 24 h. Importantly, hyperglycemic and normoglycemic subjects exhibited similar infarct sizes (Table 3).

Comparing hyperglycemic obs-AMI and hyperglycemic MINOCA patients, similar values of inflammatory parameters were detected at admission. In contrast, higher levels of WBC and neutrophils were evident after 24 h among the obs-AMI cohort. Notably, hyperglycemic obs-AMI subjects exhibited higher troponin levels, greater LVEDVs and a depressed LV function, all markers of larger infarct size (Table 3).

## Discussion

Our study was focused on the interplay between hyperglycemia, inflammation and infarct size in a cohort of patients admitted with acute myocardial infarction, including cases of MINOCA, a still poorly investigated nosological entity.

Hyperglycemia was homogeneously associated with an increase of all inflammatory indices at admission, irrespective of the underlying ischemic pathophysiological mechanism, either obs-AMI or MINOCA. Importantly, hyperglycemia correlated with the detection of large infarct sizes in patients with obs-AMI while no differences were observed between normoglycemic and hyperglycemic MINOCA cases, which exhibited a modest myocardial damage.

### Hyperglycemia and inflammation markers in obstructive-AMI

Among our overall study population, hyperglycemia was more frequently observed in patients with obs-AMI. This subgroup of hyperglycemic subjects exhibited an “inflammatory status” as expressed by increased levels of all measured inflammatory markers. High values of NLR, NPR, PLR and CRP had been previously described in this setting, and our results are in line with the existing literature, confirming the relationship between glycemic disorders and inflammation in the context of obs-AMI [22, 23]. Indeed, the activation of inflammatory mediators and pathways is vastly described as a cornerstone of atherosclerosis [24], not only in terms of chronic arterial remodelling but also favouring plaque instability and rupture [25]. Moreover, some studies have identified an association of elevated inflammatory markers, including NPR and NLR, with larger infarct sizes and an increased risk of short-term mortality [12, 13, 26].

Hyperglycemia-mediated alterations may further precipitate the atherosclerotic process [27–29]. In fact, not only does hyperglycemia amplify the inflammatory cascade, but it is also promoted by the inflammatory process itself throughout the generation of insulin-resistance and gluconeogenesis [30–32]. As a result, the interplay between hyperglycemia and inflammation triggers a vicious circle, ultimately leading to a heightened atherosclerotic burden and plaque rupture [33] with an increased mortality risk [1, 23, 34].

### Hyperglycemia and inflammatory markers in MINOCA

The main novelty of our study is that for the first time, we investigated the correlation between glycemic levels inflammatory markers in MINOCA patients. Similarly, to the results observed in obs-AMI, hyperglycemic MINOCA subjects had higher values of NLR, NPR, and PLR than normoglycemic ones.

Shared underlying pathophysiological mechanisms may explain the complex interplay between hyperglycemia, inflammation and MINOCA. In particular, a central role seems to be played by endothelial dysfunction [35]. In this setting, several studies have identified endothelial dysfunction as a determinant factor towards coronary artery vasoconstriction and vasospasm, resulting in myocardial ischemia [36]. As abovementioned, inflammation has the possibility of impairing endothelial function throughout the reduction of endothelium-derived vasodilators bioavailability, thereby decreasing the expression of endothelial nitric oxide synthase (eNOS) and nitric oxide synthesis. Another potential mechanism is the cytokine-mediated imbalance of the autonomic nervous system. Specifically, the hypothalamic–pituitary–adrenal axis response to inflammation causes an upregulation of the sympathetic system leading to coronary vasoconstriction, affecting both macro and micro-circulation [37].

Although hyperglycemia in the context of MINOCA is still largely unexplored, it seems plausible that the same mechanisms described in obs-AMI may be valid in MINOCA as well. Supposedly, hyperglycemia can further precipitate the endothelial homeostasis and amplify the inflammatory process conferring an unbalanced vascular tone and a prothrombotic state, ultimately increasing the ischemic burden [38, 39].

### Infarct size and hyperglycemia in obstructive-AMI and MINOCA patients

Hyperglycemic obs-AMI patients showed a larger infarct size than normoglycemic ones while in MINOCA no correlation was observed between admission glucose levels and the extent of myocardial damage, which was overall modest in such cases.

The link between hyperglycemia and large infarct size in the context of obs-AMI is well established, and our results are in line with previously published studies [40, 41]. In fact, several strategies have been adopted to assess the impact of admission hyperglycemia on the extent of myocardial damage, all leading toward the same conclusion [42, 43].

On the other hand, the impact of hyperglycemia on the magnitude of infarct size in MINOCA patients is still unexplored. Our findings suggest for the first time a negligible correlation between admission glucose levels and myocardial damage among MINOCA cases, who overall exhibited a limited infarct size, especially when compared to hyperglycemic obs-AMI subjects. The explanation to such results might be found in CMR studies specifically focused on the myocardial substrate of MINOCA. In particular, studies showed areas of myocardial oedema either associated with small necrotic regions with a typical patchy distribution or even without necrosis [44, 45]. Since myocardial damage observed in our cohort was minimal, it is difficult to elucidate the actual impact of hyperglycemia in the still hazy world of MINOCA. Keeping in mind that endothelial dysfunction seems to be a key pathophysiological mechanism underlying MINOCA and it is known to be impaired by hyperglycemia as well, it appears plausible that an “hyperglycemic environment” can further alter the endothelial homeostasis and negatively affect the natural history of such patients, often characterized by heart failure with preserved ejection fraction. This hypothesis clearly requires future investigations in order to evaluate whether hyperglycemia may represent a prognostic risk factor for MINOCA subjects, regardless of a concomitant diabetes diagnosis [46–51].

### Study limitations

Our study had several limitations. First of all, analyses were conducted on a relatively small sample size, especially regarding the MINOCA cohort. Second, not all laboratory parameters were available for each patient. Furthermore, it is not possible to clarify whether admission hyperglycemia has a direct relationship with infarct size and inflammatory markers or simply represents a marker of myocardial ischemia. In fact, the study does not establish causality between hyperglycemia and acute myocardial infarction by the nature of its cross-sectional design.

In patients with suspected DM, no definite rule-out criteria were adopted. However, not all patients can undergo an oral glucose tolerance test in the setting of AMI. Therefore, HbA1c could be a reasonable alternative in this clinical situation. Lastly, in our study we did not evaluate other inflammatory markers such as IL-6, TNF-α, IL-1, and the soluble matricellular protein Cysteine-rich angiogenic inducer, which might reflect the inflammatory status more accurately. Taking into account that a correlation between such parameters and the indices adopted in our study was previously demonstrated [11–14, 47, 48], we chose to use common and widely available inflammatory markers for simplicity.

## Conclusion

In patients with acute myocardial infarction, hyperglycemia was associated with a larger infarct size in obs-AMI while no differences were observed in MINOCA. Hyperglycemic obs-AMI cases presented elevated inflammatory markers both at admission and after 24 h whereas in MINOCA this data was evident only at the time of hospitalization, paralleling the modest myocardial damage detected in such patients. Our findings have pathophysiological and therapeutic implications, especially for obs-AMI subjects who can benefit from aggressive secondary therapies. Further prospective studies are needed to assess the prognostic role of hyperglycemia in the heterogenous MINOCA entity.

## Data Availability

The datasets used and/or analysed during the current study are available from the corresponding author on reasonable request.

## Abbreviations

AMI: Acute myocardial infarction
T2DM: Type 2 diabetes mellitus
MACE: Major adverse cardiovascular events
aHGL: Admission high glucose levels
NLR: Neutrophil-to-lymphocyte ratio
PLR: Platelet-to-lymphocyte ratio
NPR: Neutrophil-to-platelet ratio
MINOCA: Myocardial infarction with non-obstructive coronary arteries
Obs-AMI: Obstructive myocardial infarction
CAG: Coronary angiography
STEMI: ST-segment elevation acute myocardial infarction
LVEDV: Left-ventricular-end-diastolic-volume
LVEF: Left ventricular ejection fraction
